# Direct analysis of aberrant glycosylation on haptoglobin in patients with gastric cancer

**DOI:** 10.18632/oncotarget.14362

**Published:** 2016-12-29

**Authors:** Jae-Han Kim, Sung Hyeon Lee, Sookyung Choi, Unyong Kim, In Seok Yeo, Su Hee Kim, Myung Jin Oh, Hantae Moon, Jua Lee, Seunghyup Jeong, Min Gew Choi, Jun Ho Lee, Tae Sung Sohn, Jae Moon Bae, Sung Kim, Yang Won Min, Hyuk Lee, Jun Haeng Lee, Poong-Lyul Rhee, Jae J Kim, Su Jin Lee, Seung Tae Kim, Jeeyun Lee, Se Hoon Park, Joon Oh Park, Young Suk Park, Ho Yeong Lim, Won Ki Kang, Hyun Joo An, Jung Hoe Kim

**Affiliations:** ^1^ Department of Food and Nutrition, Chungnam National University, Yuseong-Gu, Deajeon, Korea; ^2^ GLYCAN Co., Ltd., Healthcare Innovation Park, Bundang-Gu, Seongnam, Korea; ^3^ Graduate School of Analytical Science and Technology, Chungnam National University, Yuseong-Gu, Deajeon, Korea; ^4^ Department of Biological Sciences, Korea Advanced Institute of Science and Technology, Yuseong-Gu, Daejeon, Korea; ^5^ Samsung Medical Center, Sungkyunkwan University School of Medicine, Seoul, Korea

**Keywords:** stomach neoplasm, haptoglobin, N-glycan, glycoprotein, biomarker

## Abstract

Based on our previous studies, differential analysis of N-glycan expression bound on serum haptoglobin reveals the quantitative variation on gastric cancer patients. In this prospective case-control study, we explore the clinically relevant glycan markers for gastric cancer diagnosis. Serum samples were collected from patients with gastric cancer (*n* = 44) and healthy control (*n* = 44). N-glycans alteration was monitored by intact analysis of Hp using liquid chromatography–mass spectrometry followed by immunoaffinity purification with the serum samples. Intensity and frequency markers were defined depending on the mass spectrometry data analysis. Multiple markers were found with high diagnostic efficacy. As intensity markers (*I*-marker), six markers were discovered with the AUC > 0.8. The high efficiency markers exhibited AUC of 0.93 with a specificity of 86% when the sensitivity was set to 95%. We additionally established frequency marker (*f*-marker) panels based on the tendency of high N-glycan expression. The AUC to conclude patients and control group were 0.82 and 0.79, respectively. This study suggested that N-glycan variation of serum haptoglobin were associated with patients with gastric cancer and might be a promising marker for the cancer screening.

## INTRODUCTION

Gastric cancer (GC) is the most frequently occurring malignancy in Korea, and one of the main causes of cancer death [1]. When patients are diagnosed early their 5-year survival rate is approximately 90% [2]. Unfortunately, the vast majority of GC patients present with advanced stage disease and the overall prognosis remains very poor. Although screening endoscopy and/or upper gastrointestinal series (UGIS) are widely performed in clinical settings to detect early-stage GC, to date no randomized study has been able to demonstrate a reduction in gastric cancer mortality [3].

Aberrant glycosylation is a major post-translational modification (PTM) of proteins, frequently observed in cancer cells and tissues [4–6]. In particular, a variety of cancers display changes in glycan structures of serum proteins such as IgG, α-fetoprotein (AFP), prostate-specific antigen (PSA) and haptoglobin (Hp) [7, 8]. Hp, a major acute-phase glycoprotein comprising 0.4–2.6% of total blood proteins, consists of two α- and two β-subunits whose glycosylation level changes in various types of cancer and inflammation [8, 9]. Hp is a highly sialylated glycoprotein with four N-glycosylation sites in β-chain at Asn 184, 207, 211, and 241 in β-chain [10]. It has been demonstrated that the glycosylated Hp is resulted directly from cancer itself rather than secondary to cancer-induced inflammation [11]. In order to discover novel glycan-related biomarkers, mass spectrometry (MS) has been widely used because of the advanced features including high sensitivity, stoichiometry and dynamic range [12–15]. Analysis of serum N-glycan profiles may readily allow the early diagnosis of various type of cancers, occupying a niche on the non-invasive, *in vitro* diagnosis area [7]. Recently, MS analyses of intact glycoprotein have been actively employed to determine characterization of therapeutic glycoproteins as well as to discover biomarkers associated with disease status [16–18]. Instead of glycan profiling, direct analysis of intact glycoprotein has merits such as simple, easy handling of sample preparation and time saving for analysis. This method could have thereby high potential to discover the novel cancer biomarkers with aberrant glycosylation.

Previously we explored the glycosylation of serum Hp whether the glycan alteration can be clinically relevant biomarkers for GC screening [8]. N-glycans bound on serum Hp were released enzymatically, and then the expression profile of N-glycans was monitored by nanoLC Chip Q-TOF system. As a result, we observed clear and clinically significant deviations between cancer patients and control groups suggesting the potential glycan biomarkers for GC diagnosis. Based on these considerations, we pursued the N-glycan alteration by analyzing intact Hp without releasing and collection of glycans from a protein. Beyond a fine analytical instrument, MS has been evaluated as a diagnostic platform technology that is able to directly measure the aberration of a glycoprotein with glycan heterogeneity.

## RESULTS

Clinical characteristics of gastric cancer patients and healthy controls were summarized in Table 1. The study population has 44 cancer-free, healthy controls and 44 GC patients in a random manner. Forty-one GC patients had metastatic disease, and three patients gave their serum samples prior to removal of primary tumor. All study participants were Koreans.

**Table 1:** Clinical characteristics of study participants

	Control (*N* = 44)	Gastric cancer (*N* = 44)
Age, years		
Median	48	55
Range	28 to 65	41 to 70
Gender		
Male	11	28
Female	33	16
Tumor stage		
1–3		3
4		41

Collected serum samples were first applied to immunoaffinity purification. No visual contamination was found on the SDS-PAGE after purification. In addition to serum samples obtained for the present study, commercially available serum (*n* = 7) was additionally subjected in parallel to ensure the quality of sample preparation and sequential MS analysis. The yield of purified Hp detected by quantification assay was estimated to be 100–500 μg per 450 μL of serum. Among them, 20 μg of purified serum Hp was used for the subsequent preparation of N-glycan for LC-MS analysis.

### Mass spectrometric monitoring of intact Hp

Previously we observed the significant variation in N-glycan expression on GC patients by MS profiling [8]. Though the aberrant glycosylation suggests a strong potential as a diagnostic biomarker, it requires the enzymatic release of N-glycan that necessitate a day of reaction and purification time. In the present study, glycosylation on Hp isolated by immunoprecipitation was directly monitored in a glycoprotein level without releasing N-glycan.

**Figure 1:**
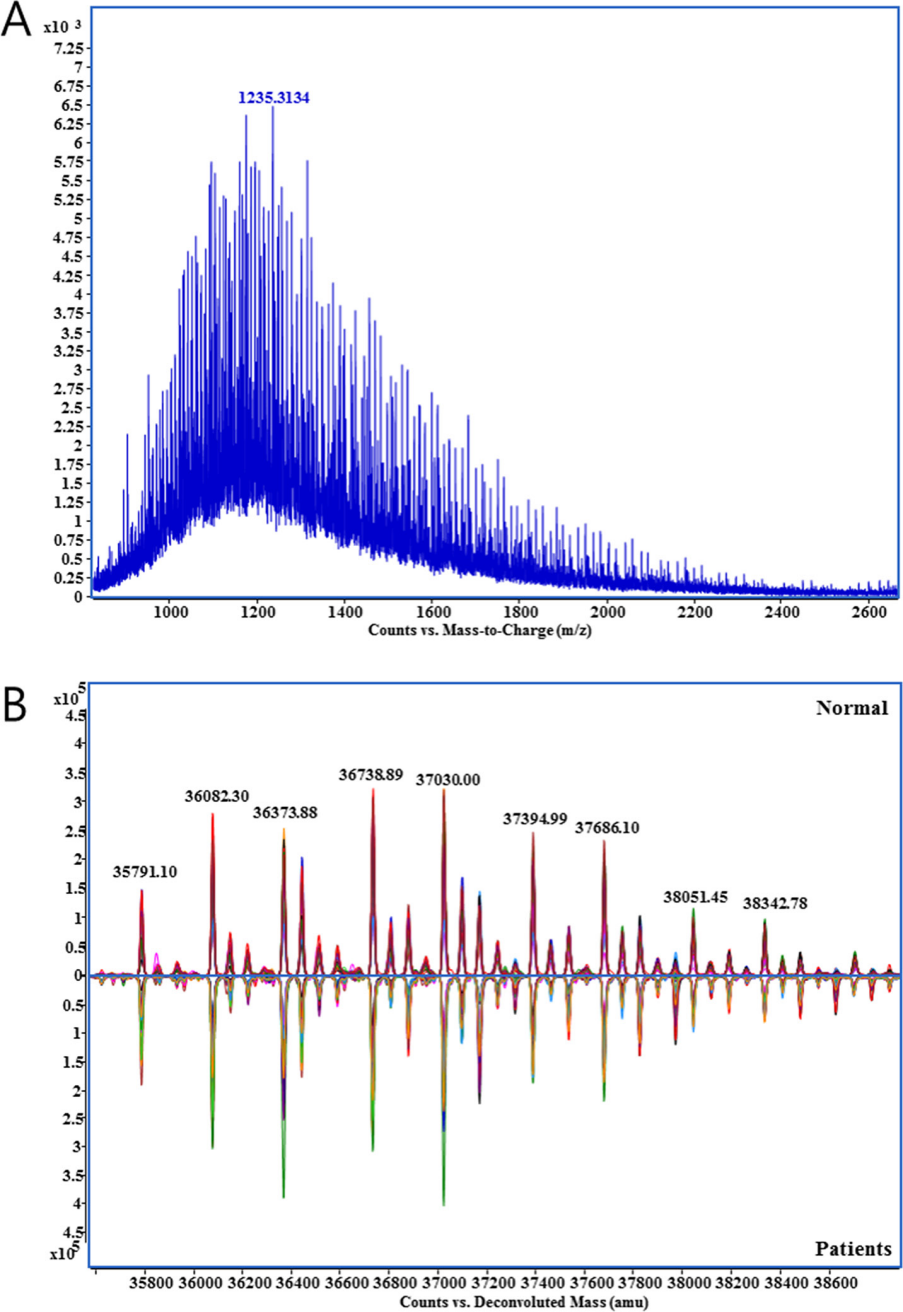
Mass spectrum obtained from the direct analysis of haptoglobin (**A**) Raw mass spectrum with multiply charged haptoglobin glycoforms., (**B**) Deconvoluted spectrum of haptoglobin glycoforms from healthy control (*n* = 44, upper) and gastric cancer patient group (*n* = 44, lower). Specta in each groups were overlaid.

Because multiple charged ion species of Hp were produced by electrospray ionization, a complex mass spectrum carrying the information on the molecular weight corresponded to each glycoform on the β-chain of Hp was generated (Figure 1A). After the deconvolution of the raw spectrum, potential glycoforms of Hp could be identified. The overlay of deconvoluted mass spectra from healthy control and GC patient serum exhibits high visual reproducibility on mass accuracy but variations on peak intensities that suggest the possibility of potential markers (Figure 1B). Peaks of glycosylated Hp were assigned by the accurate masses and glycan correlations. N-glycans heterogeneity of and correlation through carbohydrate (monosaccharide) unit are biological characteristics of protein-bound glycans [19, 20]. Human N-glycans were biologically synthesized through the competitive but sequential transfer of four unit carbohydrates, hexose (Hex), N-acetylhexosamine (HexNAc), fucose (Fuc), and N-acetylneuraminic acid (NeuAc) [21–23]. From the common core structures, specific glycosyltransferases added these unit carbohydrates, therefore, each N-glycan bound to Hp should exhibit the unique mass deviation of those four unit carbohydrates showing multiple peak from a single protein [8, 24, 25].

**Figure 2:**
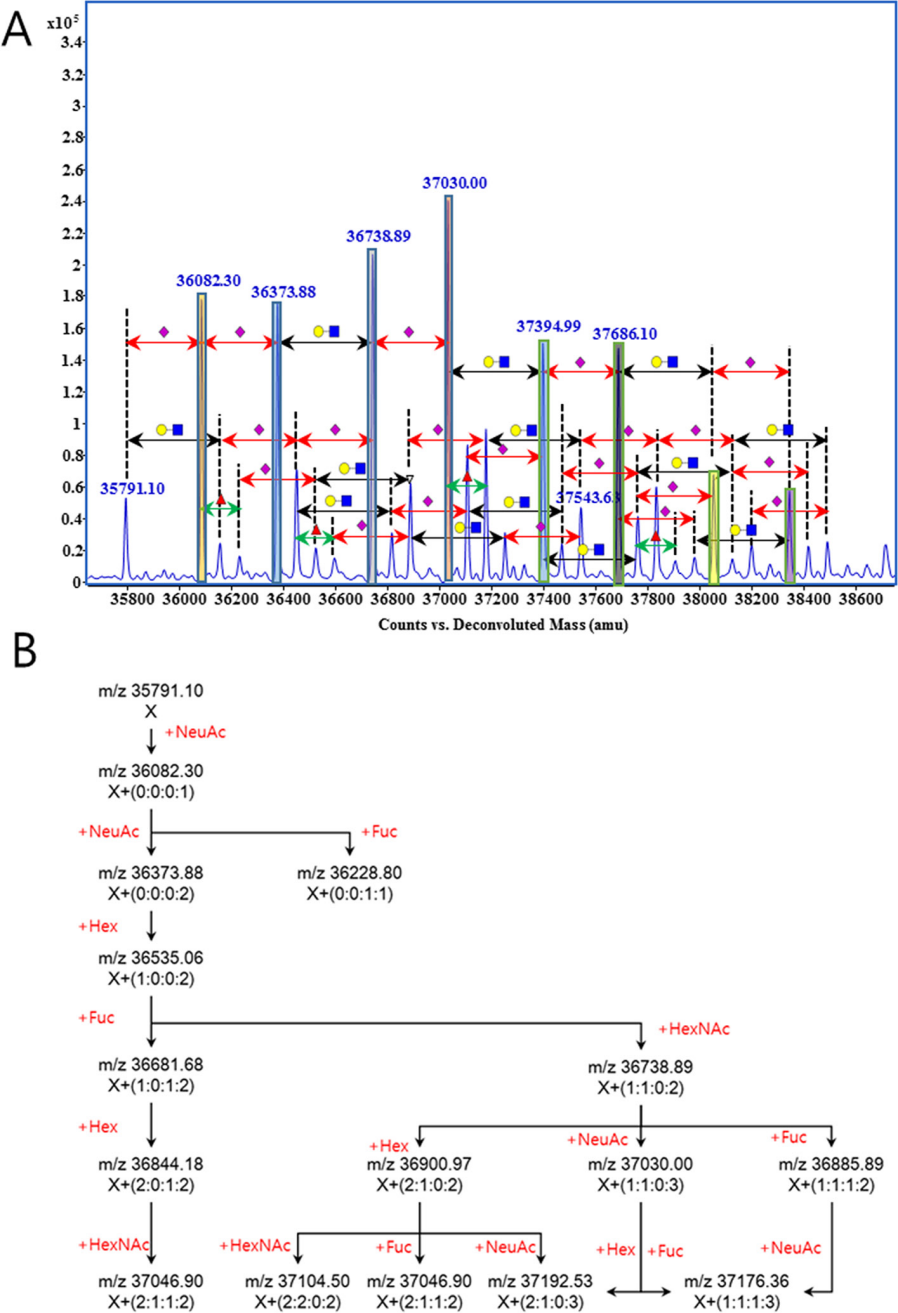
A set of glycan correlation observed in the mass spectrometric analysis of human haptoglobin Glycan correlation was displayed on the mass spectrum (**A**), and illustrated as schematic diagram (**B**). Symbols in Figure 2(A) were ●, hexose; ◼, N-acetylhexosamine; ▲, fucose; and ◆, N-acetylneuraminic acid. Number in parenthesis on Figure 2(B), are the number of carbohydrate units attached on the molecule X (*m/z* 35791.10). Number and type of units were calculated from the accurate mass and indicates hexose, N-acetylhexsamine, fucose and N-acetylneuraminic acid from left to right, respectively.

In a mass spectrum, a group of peaks having a correlation linked by the mass of each unit carbohydrate (Figure 2A) could be found. As described in Figure 2B, peak of *m/z* 35791.10 and *m/z* 36082.30 has the mass difference of NeuAc (291.25), then *m/z* 36082.30 can be connected to *m/z* 36373.88 and *m/z* 36228.80 by NeuAc and Fuc unit, respectively. As such, it could be calculated that ion molecules of *m/z* 36681.68 contain one Hex, one Fuc, and two NeuAc (glycan code-1:0:1:2) of additional glycan residues on the molecules of m/z 35791.10. These sequential correlations clearly indicate that the signals are obtained from N-glycan variation of a single protein. From seven repetitive MS analyses with standard Hp from commercial serum, we observed 74 peaks that exhibit the single step correlation of unit carbohydrate.

### Analytical stability and reproducibility

**Figure 3:**
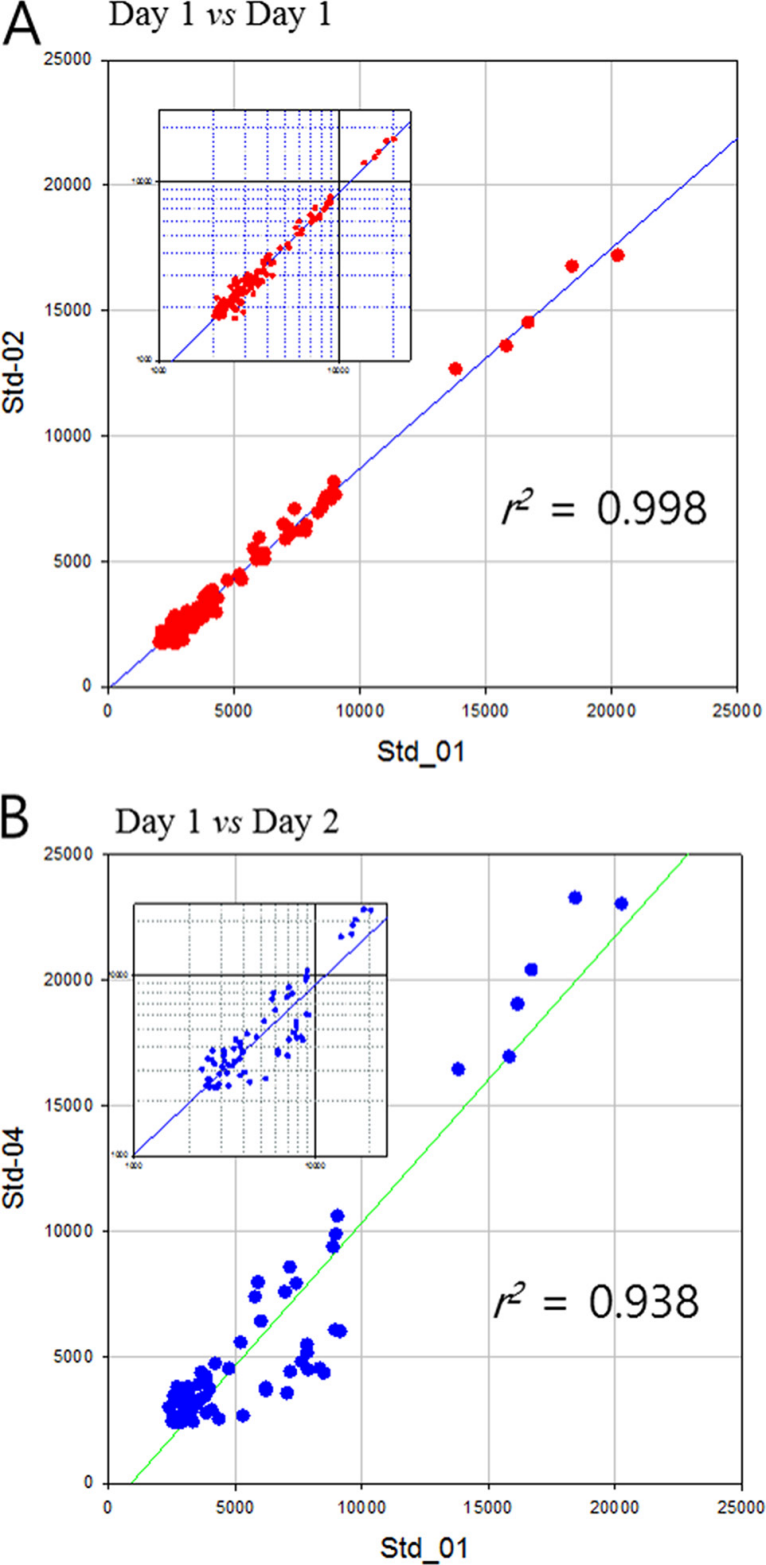
Pearson's correlation of mass spectrometric dataset of standard haptoglobin samples (**A**) analyzed same day and (**B**) different days. Small graphs are log-log plot to clarify the dispersion of small values.

Hp purified from a commercial serum was used as a quality control standard for clinical sample analysis. Each clinical sample was analyzed blindly and randomly repeated three times which made up of seven batches of sample run. More than one standard Hp from commercial sera were included in each sample batch to ensure the reproducibility and stability of analysis. As a result, MS profile of standard Hp showed extremely high reproducibility, with the range of 0.940–0.998 of a *Pearson* correlation coefficient through the whole process. Moreover, the average coefficient of variation (CV) of peak intensity in a sample was maintained under 18% indicating high quantitative reproducibility of this entire analysis process (Table 2). Daily variation of MS analyses was in acceptable ranges as well. MS profile of standard Hp analyzed in a same day showed the high reproducibility of *r^2^* > 0.99 (Figure 3A). Mass profile of standard Hp between different days of operation exhibits slight less yet still high reproducibility of *r^2^* > 0.94 (Figure 3B).

**Table 2:** Average CV of peak intensities obtained seven independent analysis of haptoglobin isolated from commercial human sera

Frequency of peak	Average CV
100~80%	18.4%
80~60%	15.9%
60~40%	15.0%

### Discovery of screening makers for gastric cancer

After collectively integrated each group's MS data, every peaks eventually have two data domains, Intensity and Frequency. Intensity represents an ion count of peak presented in each sample. Each peak has intensity values as much of its observations throughout the analysis of patient and control groups. Meanwhile, frequency (presence) of a peak indicates percentile of the sample that contains the peak among total samples resulting a single value per sample groups. Markers were found independently from these two data domains designated intensity marker (*I*-marker) and frequency marker (*f*-marker).

*I*-marker is a peak that exhibits the significant difference of their intensities between patient and healthy control groups, which is traditionally used in the diagnosis of diseases. To avoid the case of ‘N/A’ or ‘unable to diagnose’, the marker peak should show up more than 80% frequency (*f*) in both sample groups (*f*(patient) > 80% and *f*(control) > 80%). Similarly, *f*-marker is an integrative marker that could be deduced from a group of peaks. Regardless of their intensity, *f*-marker only counts the tendency (occurrence) of their presence in patient group or healthy control group.

**Figure 4:**
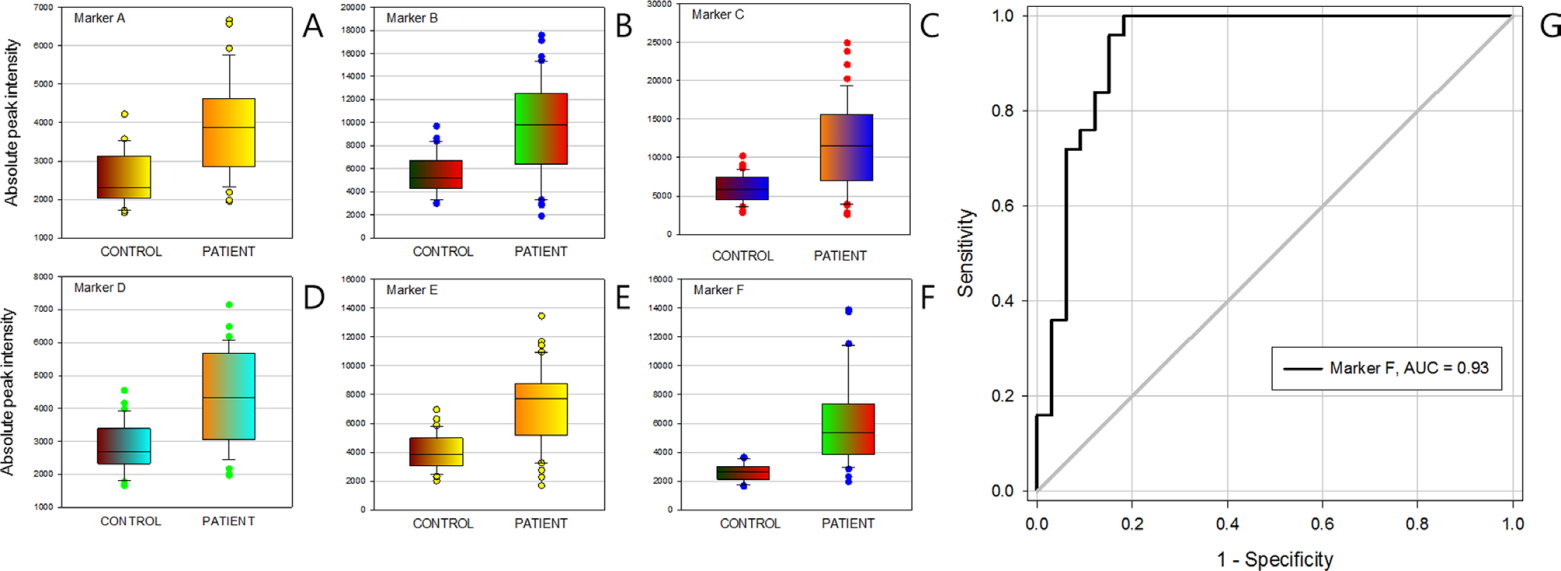
Clinical efficacy of intensity markers (*I*-marker) (**A**) to (**F**) were Box-plot of intensity marker A to marker F, (**G**) the receiver operating characteristic curve (ROC) of intensity marker (F).

To discover the *I*-markers, student's *t*-test was performed initially with the consideration of peak frequency searching potential marker candidates. Among the peaks with *P* < 10^−5^, the diagnostic markers were selected only if the AUC of ROC curve was higher than 0.80. Six screening markers were determined finally with the AUC range 0.81 to 0.93 (Table 3, Figure 4A–4F). The best predictive marker for gastric cancer was *m/z* 37978.98 corresponded to Hex_22_HexNAc_18_Fuc_2_NeuAc_10_ which showed AUC 0.93 (Figure 4G). Though the sensitivity and specificity of marker are moving along with the ROC curve line depending on the threshold values, it showed the 85% sensitivity when the specificity was fixed at 96%.

**Table 3:** Clinical efficacy of intensity markers (*I*-marker)

	*m/z*	Patient				Healthy control				*p*-value	AUC
		AVE	±	SD	CV	AVE	±	SD	CV		
Marker A	36593.422	3899	±	1257	32%	2535	±	679	27%	3.2.E-05	0.83
Marker B	36885.354	9615	±	4027	42%	5512	±	1693	31%	1.4.E-07	0.81
Marker C	37176.968	11483	±	5601	49%	5977	±	1864	31%	4.0.E-08	0.81
Marker D	37248.766	4293	±	1358	32%	2802	±	733	26%	3.6.E-07	0.84
Marker E	37541.054	7192	±	2756	38%	4048	±	1227	30%	6.6.E-09	0.83
Marker F	37978.984	6175	±	3049	49%	2585	±	598	23%	3.3.E-07	0.93

*m/z*, molecular mass; AVE, average; SD, standard deviation; CV, coefficient of variation; AUC, area under the curve.

**Figure 5:**
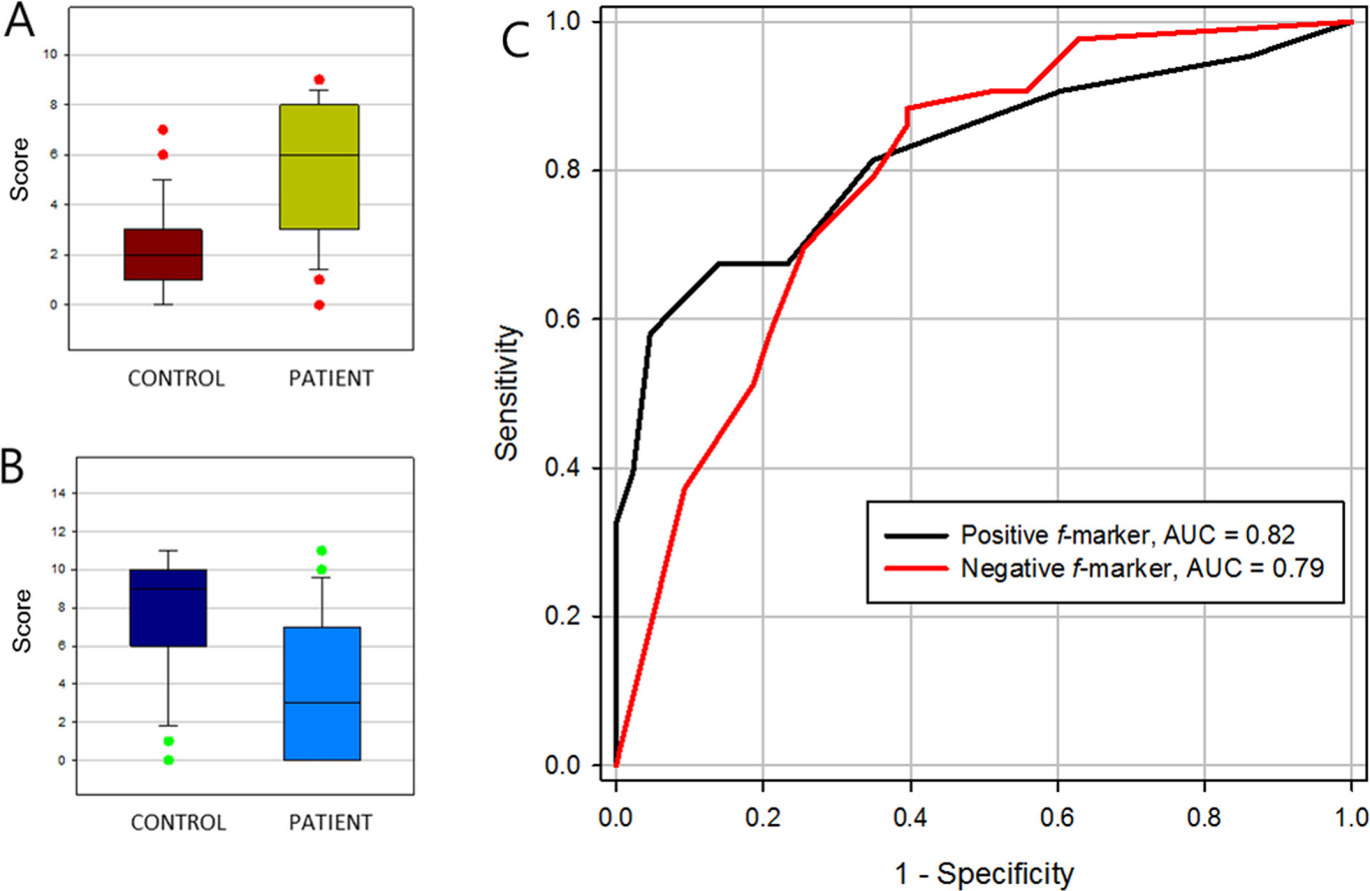
Clinical efficacy of frequency markers (*f*-marker) (**A**) positive *f*-marker, (**B**) negative *f*-marker and (**C**) their receiver operating characteristic (ROC) curve.

*f*-markers were defined as a group of N-glycans that expressed more frequently in either patient or healthy control group. Regardless of their intensity, peaks with the ratio of frequency above 1.5 fold change were collected and categorized in two groups depending on their tendency. If a group of N-glycans observed more frequently in patient group, we called it a positive *f*-marker. If a group of N-glycans had opposite trait, it was designated as negative *f*-marker. Then, a score was estimated by counting the number of peaks presence in the sample. As a result, each sample could have a positive *f*-marker value and another negative *f*-marker value. The average score (count) of positive *f*-marker in patient group was 5.4 ± 2.7, while in control group was 2.3 ± 1.8. Four negative *f*-marker, average score of control group was 7.7 ± 3.1 which was high enough to segregate the patient group having a value of 3.6 ± 3.4. The *P*-value and the AUC of ROC of positive *f*-marker were 7.81 × 10^−9^ and 0.82, respectively. Negative *f*-marker which was able to positively distinguish healthy control group exhibited the *P*-value of 6.3 × 10^−7^ and AUC of 0.79 (Table 4, Figure 5).

**Table 4:** Clinical efficacy of frequency marker (*f*-marker)

	Frequency		Frequency ratio	Count						*P*-value	AUC
	f(N)	f(P)		Control group			Patient group				
Positive *f*-marker	26%	72%	2.8								
	49%	88%	1.8								
	23%	63%	2.7								
	49%	77%	1.6								
	14%	56%	4.0	2.3	±	1.8	5.4	±	2.7	7.81.E-09	0.82
	33%	70%	2.1								
	7%	37%	5.3								
	9%	33%	3.5								
	16%	47%	2.9								
Negative *f*-marker	79%	26%	3.1								
	74%	44%	1.7								
	63%	26%	2.5								
	40%	7%	5.7								
	86%	42%	2.1								
	56%	28%	2.0	7.7	±	3.1	3.6	±	3.8	6.31.E-07	0.79
	86%	53%	1.6								
	91%	53%	1.7								
	60%	30%	2.0								
	60%	23%	2.6								
	72%	30%	2.4								

f(N), frequency in control group; f(P), frequency in gastric cancer patient group; Count, numbers of f-markers in each sample; AUC, area under the curve.

## DISCUSSION

When it comes to GC, a significant geographic variation in the incidence exists, with the highest rates being reported in East Asian countries [26], including Korea, China and Japan. In Korea and Japan, where mass screening is performed widely, early detection is often possible [27]. However, mass screening for GC, particularly by endoscopy, may not be the most practical approach because of reasons such as acceptance, availability, and cost [3]. Even in Korea where a nationwide screening program provides free-of-charge biennial endoscopy for men and women aged 40 or over [28], only 43% of the targeted population had undergone GC screening [29]. Besides gastric endoscopy, noninvasive methods for early detection of GC include UGIS and serum pepsinogen assay [29]. These screening programs have meant that patients are more likely to be diagnosed with early GC so that have it treated effectively. Unfortunately, the sensitivity of UGIS and serum pepsinogen has been found to be less than 50% in detecting early GC [29], and it is still unclear whether the screening reduces the number of people who eventually die from the disease [3].

Although the use of tumor biomarkers has been proposed for decades, the discovery of specific genetic or protein biomarkers has been fundamentally complex due to technical issues with comprehensive expression platforms, limitations in multiplex clinical assay development, and most importantly an incomplete understanding of tumor biology. The use of accurate serum biomarkers to evaluate patients with GC could, if found, lead not only to early detection but also to potential reduction in healthcare costs. Recently, a growing body of evidence supports crucial roles for glycans at a number of pathophysiological steps of tumor progression [30]. Glycans are covalent assemblies of sugars that exist in either free-form or in covalent complex with proteins or lipids, and surfaces of all mammalian cells are covered with a thick layer of complex glycans [31]. Importantly, the altered glycosylation is one of the most common and most complex PTM and plays a critical role in cancer cell biology hallmarks such as proliferative signaling, immortality, angiogenesis, invasion and metastasis [32].

Interestingly, serum measurement of certain glycans is already used to facilitate diagnosis, surveillance, or provide a surrogate measure for therapeutic outcome. For example, although the sensitivity and specificity were not very high enough to gain grade 1 recommendations [33], the serological markers CA125, CA19-9, AFP and PSA are biomolecules decorated with glycans that are commonly overexpressed by a number of cancer types. The majority of serum proteins are glycosylated, and changes in protein glycosylation are recently important focus in biomarker studies. In particular, serum Hp, an acute-phase glycoprotein, is highly expressed in a variety of cancer types [34], but its glycosylation status is different from one type of cancer to another [35]. Previous reports demonstrated that original tissue and cell producing aberrant glycosylation of serum Hp is different in each cancer type [9, 11, 36, 37]. In GC, a previous study showed the increased level of SLe^x^ in serum Hp based on western blotting analysis with anti-SLe^x^ antibody [38]. Furthermore, our previous study showed that aberrant glycan structures of fucosylation linked to SLe^x^ epitope in GC that was revealed by N-glycan profiling base on LC-MS analysis [8].

Technical advances in analytical methodology have greatly enhanced our capabilities for analysis of human glycans, although inherent diversity and complexity make analytic process extremely difficult. Especially, MS is emerging as a powerful technique for glycan profiling and structural elucidation, and can be used as a precise tool to facilitate identification of aberrant glycosylation related to cancers [12, 39, 40]. It was found that, by MS analysis, GC patients have distinctive serum N-glycan patterns including the high-mannose-type glycans, glycans with one complex type antenna, bigalactosylated biantennary glycans, and nongalactosylated biantennary glycans [41]. However, most of previous studies utilized glycan itself released from carrier molecules (*e.g*., protein, lipid, other organic compound) for MS analysis, a time consuming process to release glycans in sample preparation because it needs an enzymatic reaction overnight. In the present study, we applied a MS technique focused on intact molecular weight of specific glycoprotein to solve the problems. It is not necessary to release glycans from human biomolecules in case of the MS analysis for intact glycoprotein, which makes the method to be able to use clinically with several benefits such as simple, easy handling of sample preparation and time saving for entire analysis.

In the present case-control study, we attempted to explore differential expressions of Hp N-glycan in GC by intact MS analysis, and to evaluate the diagnostic performance of N-glycan variation of intact Hp as a potential screening marker for GC. To identify candidate glycan markers on intact Hp, we initially focused on intact *m/z* signals which were quantitatively strong enough to distinguish the patients and healthy control groups and whose isotopic peak distribution was apparent in all samples. The diagnostic efficacy based on *P*-values and AUC was evaluated in 88 serum samples (44 healthy controls and 44 patients) that had been randomly selected. Conclusively, we found more than a few aberrant glycan markers bound to serum Hp that have high sensitivity as well as high specificity in differential diagnosis of GC patients and healthy controls. One may argue that the accuracy of a screening test is indicated by its sensitivity and specificity. Our novel glycan biomarkers have high sensitivity as well as specificity. Furthermore, when combined together, the AUC of the glycan biomarkers reached as high as AUC 0.8 ~ 0.93. Because GC is considered a heterogeneous disease, it seems unlikely that one biomarker will be uniformly elevated. Therefore, a panel of biomarkers (i.e., multi-biomarker) may provide more accurate diagnosis than any given marker alone. To the authors’ best knowledge, this is the first and only study to prove a diagnostic value of aberrant glycans bound to serum Hp based on intact molecular weighting in cancer patients.

Our findings have limitations, and warrant further validation studies including in a larger study as well as in patients with other cancer types than GC. Our results showed a disparity in baseline characteristics between GC patients and control, although studies suggested glycomic profiling may be dependent on the subjects’ age and gender [42]. A majority of GC patients had an advanced stage disease. The small sample size is also inadequate to make any definitive conclusions on the performance of our finding, so the validation study involving a larger number of samples is warranted. In addition, because the sample lacked diversity with respect to ethnic group, it is unclear the finding should also be applied to other race than Korean. When it comes to the prognosis of patients with GC, although randomized clinical trials showed similar magnitude of survival benefit with chemotherapy [43], it is known that survival outcome and tumor biology may differ considerably between Western and Asian countries. However, the results of the present study provide evidence for serum measurements for aberrant glycans bound to serum Hp in early detection of GC, although the mechanism underlying the alterations in glycans has not been fully understood yet. Moreover, the role of these aberrant glycosylations in relation to prognosis in GC is outside the scope of this study, and remains as a topic for extensive further studies. Further studies focused on glycan alterations in cancer cells or tissues may help to identify cancer-specific glycan pathways. In addition to the role of glycans as cancer biomarkers [44], the broad spectrum of glycans that activate cancer proliferation, invasion and metastasis may lead to a range of glycan-specific targeting approaches for novel drug development [45]. Our results provide further insight into the glycomics based on MS screening in cancer development, and may provoke more detailed investigations leading to identification of a panel of diagnostic serological biomarkers applicable to early detection of cancer.

## CONCLUSIONS

We studied whether N-glycan variation in serum Hp based on intact *m/z* signals is occurred and showed high efficiency diagnostic values as screening markers for GC. Despite a small study with only 88 samples tested, we might conclude that aberrant glycans bound to serum Hp are associated with patients with gastric cancer and are a potential novel marker for gastric cancer screening. Further, multiple biomarkers with high diagnostic efficacy might enable the biomarker panel test, results of which can be obtained from a single MS analysis.

## MATERIALS AND METHODS

This was a prospective case-control study, in which a total of 88 serum samples were obtained from 44 patients with GC and from 44 cancer-free healthy volunteers (Table 1) at Samsung Medical Center (SMC, Seoul, Korea). All serum samples were collected and processed in an identical fashion and the cancer and control samples were obtained over the same study periods. This study protocol (ClinicalTrials.gov identifier: NCT02848066) was reviewed and approved by the SMC Institutional Review Board (IRB# SMC2015-07-146-001). All participating subjects provided written informed consent in accordance with the Declaration of Helsinki, after having been informed about the purpose and investigational nature of the study.

### Immunoaffinity purification of serum Hp

Serum Hp purification was performed using Hp antibody-conjugated affinity column [11]. In brief, 450 μL serum from each subject was diluted in 4.5 mL phosphate-buffered saline (PBS, pH 7.4), and then applied to the anti-Hp affinity column. After washing the unbound components with 30 mL PBS and elution of Hp with elution buffer (0.1 M glycine, 0.5 M NaCl, pH 2.8), and the elutent was fractionated into a tube containing neutralization buffer (1.0 M Tris-HCl, pH 9.0). The detergent was removed and eluent samples were concentrated using a centrifugal filter (MWCO 10,000, Amicon Ultra, Millipore; Billerica, MA, USA). Quantification assay of purified Hp was performed using a Quant-iT Assay Kit (Invitrogen; Carlsbad, CA, USA). To check the purity of Hp, the eluent was randomly applied to 12.5% SDS-PAGE with Coomassie Brilliant Blue staining. Each sample was lyophilized and kept at −80°C until analysis.

### Denaturation and alkylation of Hp

Twenty micrograms of purified Hp were dissolved in a buffer, which consisted of 50 mM NH_4_HCO_3_ (pH 9.0), 5 mM DTT, and 14 mM IAA. The Hp dissolved in buffer solution was boiled in water for 10 min at 90°C to reduce the disulfide bridges in α- and β- subunits of Hp, and reduced sulfhydryl groups were alkylated with IAA to prevent reconstruction of disulfide bridges. Finally, denatured Hp was evaporated in vacuum, and then reconstituted with 200 μL of distilled water. One microliter (100 ng of Hp) was injected into the nanoLC-chip/Q-TOF MS system.

### NanoLC-chip/Q-TOF MS analysis of Hp

The analytical chip (Agilent Technologies Inc., San Jose, CA, USA) consisted of 9 × 0.075 mm enrichment column and 43 × 0.075 mm analytical column both of which were packed with 5 μm C8-bonded particles. A rapid elution gradient for Hp was delivered to the analytical column at 300 nL/min using mobile phases of (A) 0.1% formic acid (v/v) in water, and (B) 0.1% formic acid (v/v) in acetonitrile, ramping from 3% to 25% B over 1 min, from 25% to 60% B over 3 min, from 60% to 80% B over 4 min, and from 80% to 95% B over 4 min. The columns were flushed out with 95% B prior to re-equilibration.

Following chromatographic separation, Hp was ionized by chip-integrated nano-ESI spray tip and analyzed by a Q-TOF mass analyzer (model 6540, Agilent Technologies Inc., San Jose, CA, USA). MS spectra were acquired in positive ionization mode over mass range of *m/z* 500 to 4500 with a scan rate of 1.5 spectra/sec. After data acquisition, raw LC/MS data was deconvoluted by maximum entropy algorithm using MassHunter software equipped with BioConfirm add-on (Agilent Technologies Inc., San Jose, CA, USA).

### Statistical analysis

Glycan correlation between peaks was calculated based on the average mass from seven independent analyses of Hp. Unit mass of hexose (162.14), N-acetylhexosamine, (203.19), fucose (146.14), and N-acetylneuraminic acid (291.25) was used. Mass tolerance for the assignment of carbohydrate correlation was less than 25 ppm. Student *t*-test was used for comparison between patient and healthy control group to screen potential markers with the assumption of two-tailed and equal variance. Area under the curve (AUC) of receiver operating characteristic (ROC) curves was calculated by the trapezoidal rule. Data handling, descriptive and statistical analyses were carried out by Microsoft Excel 2013 (Microsoft, Seattle, WA, USA), SigmaPlot 12.5 (Systat Software Inc., San Jose, CA, USA) and Stata 12 (Stata Corp., College station, TX, USA).
